# Investigation of the scattering and attenuation properties of cataracts formed in mouse eyes with 1060-nm and 1310-nm swept-source optical coherence tomography

**DOI:** 10.1364/BOE.433927

**Published:** 2021-09-20

**Authors:** Pablo Eugui, Conrad W. Merkle, Johanna Gesperger, Antonia Lichtenegger, Bernhard Baumann

**Affiliations:** 1 Center for Medical Physics and Biomedical Engineering, Medical University of Vienna, Austria; 2 Division of Neuropathology and Neurochemistry, Department of Neurology, Medical University of Vienna, Austria

## Abstract

Cataracts are the leading cause of blindness worldwide. Here we propose optical coherence tomography (OCT) as a quantitative method for investigating cataracts. OCT provides volumetric and non-invasive access to the lens and makes it possible to rapidly observe the formation of opacifications in animal models such as mice. We compared the performance of two different wavelengths – 1060 nm and 1310 nm – for OCT imaging in cataract research. In addition, we present multi-contrast OCT capable of mapping depth-resolved scattering and average anterior cortical attenuation properties of the crystalline lens and quantitatively characterize induced cataract development in the mouse eye. Lastly, we also propose a novel method based on the retinal OCT projection image for quantifying and mapping opacifications in the lens, which showed a good correlation with scattering and attenuation characteristics simultaneously analyzed during the process of cataract formation in the lens.

## 1. Introduction

Affecting 20 million people, cataracts are the leading cause of blindness worldwide [1,2]. Cataracts are caused by the formation of opacifications in the ocular lens, an ellipsoid structure located in the anterior part of the eye [3,4]. A transparent tissue in healthy eyes, the cataractous lens becomes opacified by microstructural changes that cause light to be scattered as it propagates to the retina, resulting in a loss of vision. Although events such as an injury of the lens or medical conditions such as diabetes can cause cataracts, most develop due to aging and affect people over 75 years of age [5,6]. Pharmacological therapies to reverse and cure the effect of cataracts are being investigated, however the standard treatment is a surgery where the opaque crystalline lens is replaced by an artificial intraocular lens [7–10].

The most common method for cataract diagnosis is a slit lamp examination where the patient’s eye is illuminated with a bright thin sheet of light to look for opacifications with a microscope [11,12]. The examiner then compares the slit lamp images to an established catalogue scheme, such as the Lens Opacification Classification System (LOCS III) [12,13], to determine the severity and type of cataract. Although this clinically established method has been the most widely used by ophthalmologists for years in addition to the visual acuity test, the results are subject to the perception of the examiner and do not provide volumetric or quantitative information [14–16]. Alternative imaging methods such as Scheimpflug, ultrasound and X-ray imaging have been proposed for clinical cataract diagnosis [17–20], but have not been able to overcome some limitations such as the sacrifice of axial resolution when scanning a large field of view with long depth range, the need for contact between probe and eye, the use of ionizing radiation or the lack of accessibility to a high-resolution X-ray scanner for many ophthalmologists that have prevented their expansion into the cataract study environment.

Optical coherence tomography (OCT) is a non-invasive imaging technique that provides a three-dimensional visualization of tissue morphology [21–22]. Using a low-coherence light source, OCT achieves imaging with micron-scale resolution by means of an interferometric detection of light backscattered from the sample. As OCT can access the semi-transparent composition of the eye in a rapid and non-invasive way, OCT has become a clinical standard for diagnosis of ocular diseases [23]. The capabilities of OCT for imaging the anterior segment of the eye have been demonstrated [24], including studies to visualize cataracts in the crystalline lens [25–33]. We recently reported a study showcasing the benefits of using OCT to investigate cataracts in mouse models [34]. However, extracting quantitative parameters about the extent of the lesion was challenging because of the different shapes of the opacifications. OCT data based assessment of the attenuation coefficient has been proposed to differentiate dissimilar tissues and provide quantitative tissue information in OCT applications such as the investigation of retinal nerve fiber layer degradation in glaucoma, the detection of cancerous tissue infiltrations or corneal transparency objective assessment [35–39]. Since lens opacifications typically decrease light transmission towards the retina, changes in the lens attenuation coefficient can be expected.

Optical tissue characteristics such as scattering and attenuation usually depend on the wavelength used [40]. In our previous study [34], we used a laser source centered at 1310 nm and achieved good light penetration through opacifications, however the scattering signals from lens portions with only mild cataract could barely be observed. While spectrometer based OCT using 840-nm light may provide stronger scattering signals, it typically lacks the ranging depth due to subpar sensitivity roll-off compared to swept-source OCT [41,42]. Swept-source OCT at 1060 nm might be a worthwhile alternative to 1310 nm and takes advantage of higher backscatter coefficients at the cost of reduced penetration.

In this article, we compare OCT imaging for two near-infrared regions commonly used for swept-source OCT [43], namely the 1060-nm and 1310-nm bands, and investigate scattering and attenuation properties of cataracts. Since most OCT investigations of cataracts reported to date have focused on visualizing lesions at the time when opacifications have already formed [25–33], we here exploit the non-invasive capabilities of OCT for longitudinal small animal imaging to investigate cataract formation over time. Finally, we also present how cataract severity directly affects retinal illumination and correlates with the attenuation and scattering measurements in the affected lens.

## 2. Methods

### 2.1 Dual-wavelength range optical coherence tomography

**Fig. 1.**
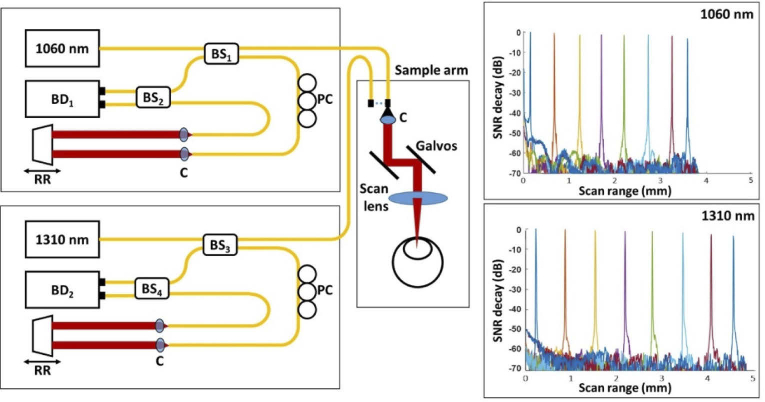
Experimental OCT setup combining the 1060-nm and 1310-nm sub-systems with a common sample arm. BD - balanced photo detector, BS_1_ and BS_3_ are 75/25 fiber beam splitters/couplers, BS_2_ and BS_4_ are 50/50 fiber beam splitters/couplers, C - collimator, PC - polarization control paddle, RR - retroreflector. The imaging range with the signal-to-noise ratio (SNR) roll-off for the respective wavelength modalities can be seen on the right.

A swept-source OCT system was built for imaging in the 1060-nm and 1310-nm wavelength bands. The two fiber-based sub-systems shared a common sample arm as depicted in Fig. 1 in order to keep the imaging setting as similar as possible and enable a direct comparison of the two wavelength bands for imaging cataracts in mouse eyes. The two OCT sub-systems were based on Mach-Zehnder interferometer configurations using a short cavity wavelength tunable laser centered at 1060 nm with a 10-dB bandwidth of Δλ* *= 100 nm (Axsun Technologies, Inc.; *1060 nm SS-OCT laser engine*) and 1310 nm with a 10-dB bandwidth of Δλ* *= 140 nm (Axsun Technologies, Inc.; *1310 nm SS-OCT laser engine*), respectively. The repetition rate was 100 kHz with a duty cycle of ∼ 50% for both light sources. The light emitted by the laser sources was divided by fiber beam splitters with a splitting ratio of 75/25 denoted as BS_1_ (Thorlabs, Inc., TW1064R3A2A) and BS_3_ (Thorlabs, Inc., TW1300R3A2) in Fig. 1, delivering 75% to the reference arms and 25% to the common sample arm. In order to achieve the most similar imaging performance possible for comparing the imaging capabilities at both 1060 and 1310-nm wavelengths and keep the eye alignment position constant, a common sample arm for both systems was constructed. The sample arm consisted of a fiber collimator (C) with f = 11.2 mm focal length launching the beam through x-y galvanometer scanners (Thorlabs, Inc., GVS002) and a 50-mm focal length scanning lens whose focus was set at the mouse lens. To switch between the two OCT modalities, the respective sample arm fiber was plugged into the collimator. This manual process took about 6 seconds. After illuminating the sample, the light backscattered traveled back through sample arm and BS_1,3_ and was guided to a 50/50 fiber beam splitter indicated by BS_2_ (Thorlabs, Inc., TW1064R5A2B) and BS_4_ (Thorlabs, Inc., TW1300R5A2) in Fig. 1. Here, it interfered with the beam coming from the reference arm. Each system had an independent reference arm featuring a retroreflector (RR) mounted on a translation stage that was adjusted to match the reference and sample arm path. A polarization paddle controller was integrated in order to match the reference and sample arm polarization and thus maximize the interference signal. The interference signals were then detected by the respective balanced photo detectors (BD_1_ and BD_2_, Thorlabs, Inc., PDB435C) with a 350 MHz bandwidth and digitized with a variable frequency of up to 500 MHz at 12-bit resolution by a high-speed data acquisition board (Alazar Technologies, Inc., ATS9360) using the respective k-clock signals of each laser. The illumination power measured at the sample was ∼ 2*.*2 mW (1060 nm) and ∼ 2*.*5 mW (1310 nm). A sensitivity of 100 dB was measured for the 1060 nm system and 98 dB for the 1310 nm system. The image depth range and signal roll-off were measured by placing a mirror at the sample position and shifting the reference arm length in intervals of 0.5 mm, obtaining the graphs shown in Fig. 1. The image depth range was 3.8 mm in air with a sensitivity roll-off of 3.7 dB for the 1060-nm sub-system and 5 mm imaging range with a roll-off of 4 dB for the 1310 nm sub-system as shown in Fig. 1. The axial resolutions of the systems were measured to be ∼ 6 µm (1060 nm) and ∼ 6*.*5 µm (1310 nm) in air corresponding to 4.2 µm and 4.5 µm in lens tissue assuming group refractive indices of 1.42 and 1.44 [43–45]. The lateral resolution for both wavelengths was determined to be ∼15 µm using an USAF resolution test target.

### 2.2 OCT data acquisition and processing

Three-dimensional data sets consisting of 768 × 512 × 512 pixels (z,x,y) were acquired with both wavelength modalities spanning a field of view of 3*.*8 × 4 × 4 mm (1060 nm) and 5 × 4 × 4 mm (1310 nm). A custom-made program (National Instruments, LabVIEW 2015) was used for driving the imaging system and acquiring the data. The post-processing pipeline was programmed in Matlab (MathWorks, MATLAB R2020b) including background removal by median spectrum subtraction, numerical dispersion compensation [47] and FFT. Due to the different wavelength ranges and the use of different optical fibers, software dispersion compensation had to be performed independently for each sub-system. Laser trigger jitter effects and motion artifacts were corrected by a custom-made software that cross-correlated and realigned consecutive B-scans.

### 2.3 OCT image analysis

**Fig. 2.**
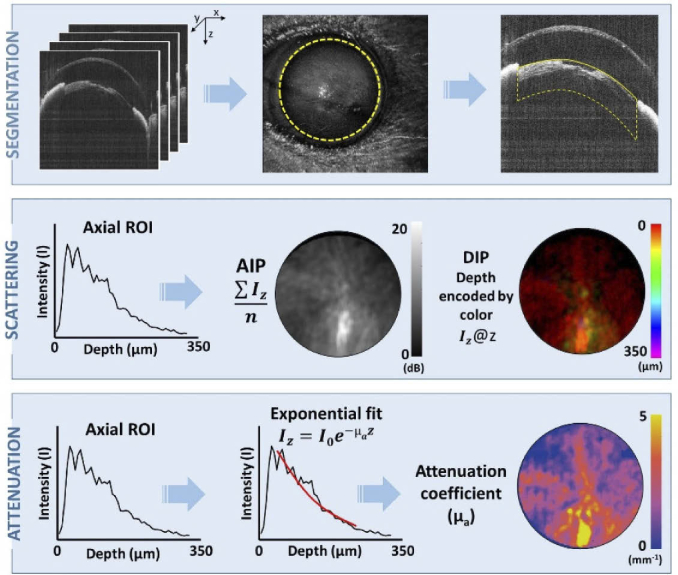
Scheme of the post-processing pipeline. A semi-automatic segmentation was used for isolating the ROI in the crystalline lens to be investigated. From this region, average intensity projection (AIP) and depth intensity projection (DIP) maps were calculated. The attenuation coefficient (µ_a_) was calculated by fitting an exponential function to the axial linear intensity data.

In order to extract the cataract features, an image processing pipeline was created. First, screening of the data sets was performed to ensure that the images were not corrupted by acquisition errors or artifacts caused by motion or shadows. A circular region of interest (ROI) representing the pupil location was then manually fitted in the axially averaged en-face image, as illustrated in Fig. 2. This ROI was used as a mask to discard the parts that were out of the pupil area. The anterior lens surface was segmented using an edge detection algorithm, followed by a 3D median filter (5 × 5 × 5 voxels) and a surface fitting function in order to interpolate gaps and areas where the lens surface reflection was barely visible, especially in eyes without cataracts. After segmenting the lens, the first 15 µm underneath the segmentation line were ignored to avoid artifacts produced by high intensity peaks produced by reflection in the lens surface. In addition, if an extreme saturation was observed during the screening of the data sets, this area from the dataset was masked and discarded of calculating the optical parameters. Once the lens surface was segmented within the pupil region, a slab covering 350 µm in depth (in tissue) was selected as the lens region for further analysis. This is illustrated in the first row of Fig. 2.

The segmentation of the lens ROI enabled to extract scattering and attenuation parameters in order to investigate the cataract. The average intensity projection (AIP) was calculated as the mean signal within the slab of the selected axial profiles and expressed in SNR as:

(1) $$SNR(dB)=20\cdot \log\left(\frac{I_{samp}}{\sigma_{bg}}\right)$$

where $I_{samp}$ is the average intensity within the selected depth range after the background signal has been subtracted and $\sigma_{bg}$ represents the standard deviation of the averaged background signal outside the pupil area in the same depth range. Depth intensity projection (DIP) images were also created by color-coding the axial location at which the pixel with maximum intensity was found, providing circular scattering maps of the eye as shown in Fig. 2. When assuming a single-scattering model, the light intensity propagating though a medium can be described as [48]:

(2) $$I(z)=\;I_{0}e^{-\mu_{a}z}$$

A widely used method for extracting the attenuation coefficient µ_a_ in OCT data is to fit a curve to the axial intensity profile and measure how rapidly light decays over the selected range scaled by the refractive index [35,49]. Nonlinear least squares curve fitting was applied to the axial profiles within a range of 350 µm in the anterior lens cortex, selected from 15 µm beneath the lens surface. Only pixels above a 9 dB SNR threshold were considered for the fit to avoid the noise influence. In A-scans with too few samples meeting the SNR threshold criterion, the attenuation coefficient µ_a_ was set to zero. The free running parameter µ_a_ in Eq. (1) was considered as the global attenuation coefficient and displayed in 2D maps for each eye as shown in Fig. 2.

The long depth range extending over the whole eye length provided by the used wavelengths enabled OCT imaging from the anterior segment to the retina in the very same data sets. However, since a telecentric scan geometry was used at the cornea, a simultaneous wide-field scan of anterior eye and retina was not possible. Note that none of the images shown in this article were corrected for refraction. The retinal OCT data represent a projection of the beam refracted at each of the anterior eye structures. Hence it is important to understand that all points in the image correspond to the same physical point in the retina (assuming an emmetropic eye) and do not represent an area of the proper retina but a transmission map of the crystalline lens. Although the focus was set at the anterior lens pole, the retinal projected layers were visible in eyes without lens opacifications and enabled the retrieval of maps representing the retinal illumination. Retinal projection maps were created by computing the average intensity over the retinal region of the OCT scans spanning 350 µm in depth. To quantify how much light was being transmitted through the lens during the cataract formation, the optical density was calculated as:

(3) $$OD=\;\log_{10}\frac{I_{0}}{I}$$

where $I_{0}$ represents the intensity of the retinal projection at the initial measurement before the cataract and *I* is the intensity measured at each specific time point.

Statistical analysis was performed by creating correlation plots and calculating the Spearman’s correlation coefficients among the different pairs of variables – namely lens scattering, attenuation and retinal SNR – by least-squares fitting of the data.

### 2.4 Animals

A group of mice (n = 4, 8 eyes total) was used to investigate cataract development in vivo and compare OCT imaging for the two wavelength ranges. Animals were kept under controlled conditions at the Center for Biomedical Research, Medical University of Vienna. The mice were anesthetized by an intraperitoneal injection of 100 mg/kg body weight ketamine and 6 mg/kg xylazine in saline (3%_vol_ xylazine, 10%_vol_ ketamine, 87%_vol_ isotonic saline). The administration of this combination also causes acute reversible lens opacities in rats and mice [50,51]. In this work, that side effect of the anesthetic was exploited as a model for cataract formation. Anesthetized mice were kept warm using a heating pad and placed in a custom-made platform that enabled a precise alignment of the eye position with respect to the OCT device. Tropicamide drops were applied to both eyes for pupil dilation. Artificial tear drops were repeatedly applied to non-scanned eye to delay cataracts. The order of wavelengths used for imaging were alternated for each mouse to eliminate potential confounds as well as which eye (left/right) was chosen to start with. After the imaging session, the animals were sacrificed by cervical dislocation. Finally, the eyes were carefully enucleated and immediately fixed in 4% formaldehyde for ex-vivo OCT analysis and long-term storage for future ex vivo investigations. The experiments were conducted as indicated by the ARVO Statement for the Use of Animals in Ophthalmic and Vision Research and Directive 2010/63/EU. The study protocol was approved by an ethics committee and by the Austrian Federal Ministry of Education, Science and Research (BMBWF/66.009/0272-V/3b/2019).

## 3. Results

### 3.1 Multi-contrast OCT imaging of cataracts

**Fig. 3.**
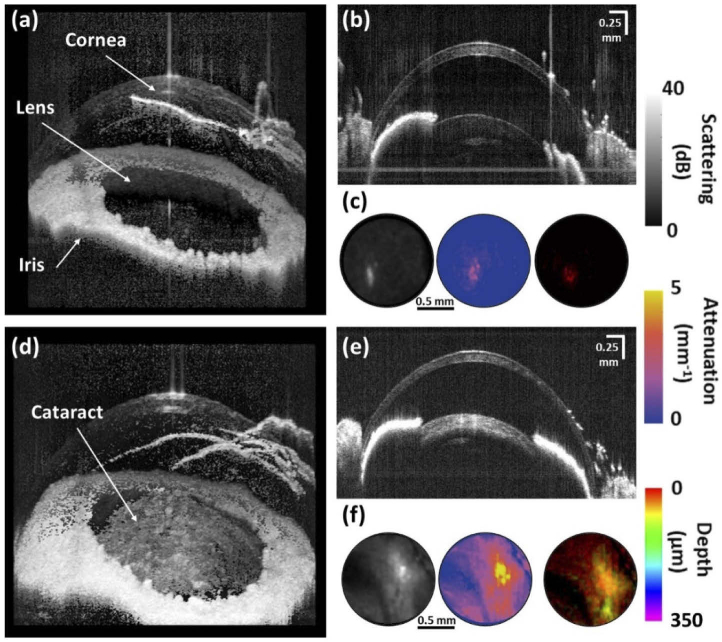
Visualization of cataracts in the mouse eye with OCT. (a-b) Images of the anterior segment of a mouse eye without cataracts. (c) Corresponding scattering, attenuation and depth projection maps of the investigated eye. (d-e) Images of the anterior segment of the same mouse eye 20 minutes later with a cataractous lesion in the crystalline lens. (f) Scattering, attenuation and depth projection maps of the corresponding eye with cataract. All images were taken at 1060 nm.

The different image contrasts provided by the custom-made dual-wavelength OCT device were used for visualizing cataracts in the in vivo mouse eye. Figure 3(a-b) show images acquired at 1060 nm of the anterior eye segment of a mouse with no opacification in the lens. Apart from the reflection at the aqueous-lens boundary, a clear lens with only very weak scattering signals can be observed. The respective scattering, attenuation and depth intensity projection maps shown in Fig. 3(c) indicate very low values with an average SNR of ∼6 dB and µ_a_ of ∼1 mm^-1^ within the evaluated ROI of the crystalline lens. Figure 3(d-e) show OCT images acquired at 1060 nm of a mouse anterior segment with an opacification in the lens. An increased scattering signal can be observed in the lens as indicated by the arrow in panel (d). The corresponding scattering, attenuation and depth intensity projection maps shown in Fig. 3(f) reveal higher average SNR and attenuation coefficient values of 23 dB and 4.2 mm^-1^, respectively, within the lens compared to the eye without opacification.

### 3.2 1060 nm vs. 1310 nm for cataract imaging

**Fig. 4.**
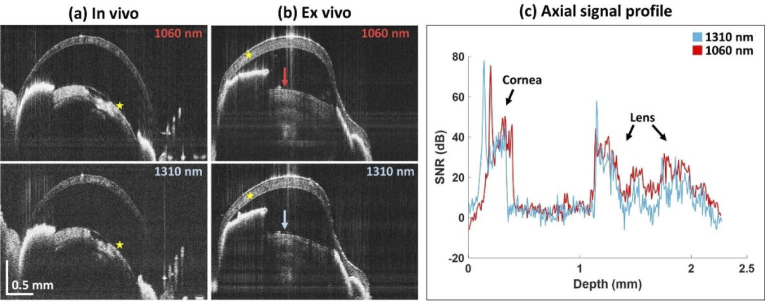
(a) Comparison of two mouse eyes imaged in vivo with OCT at 1060 nm and 1310 nm. (b) Comparison of OCT images for the respective wavelengths acquired ex vivo. (c) The depth profile of the eye in the position indicated by the arrows is shown. Note that the depth is expressed in optical path and scaled by the average lens refractive index. A higher scattering signal from the crystalline lens can be observed for OCT data acquired with the 1060-nm sub-system. The small difference in eye lengths for each wavelength might be caused by a shrinking of the ex vivo eye due to dehydration, a mismatch between scanning coordinates due to the different optical behavior of each wavelength as it passes through the scanning lens of the system and/or a difference in the refractive index of aqueous and cornea for the respective wavelengths.

OCT imaging at 1060 nm and 1310 nm was performed in the anterior segment of different mice. When comparing the OCT images acquired in vivo at 1060 nm and 1310 nm (see Fig. 4(a)), both wavelength bands were capable of visualizing opacifications in the rodent eye. However, a slightly higher scattering signal with the average SNR increased by ∼8 dB within the opacification was observed in the 1060 nm data sets compared to 1310 nm. The increased SNR resulted in better contrast of the opacifications and a more uniformly distributed signal within the structures such as cornea or lens opacification as indicated by the yellow markers in Fig. 4. However, in several cases the image quality provided by the 1310 nm sub-system was similar to or even better than the 1060 nm, potentially caused by a mismatch in the focus position or by small movements of the animal. In order to have a one-on-one comparison using the exact same alignment for both modalities, we also performed an ex vivo comparison with a cataractous eye that was enucleated after the imaging session. The results are shown in Fig. 4(b). A slightly higher and more homogeneous signal can be observed within the lens opacification for 1060 nm OCT compared to the 1310 nm image. The axial OCT profiles of this eye at the location indicated by arrows in the tomograms are shown in Fig. 4(c) where a more pronounced scattering signal in the opaque lens portion can be observed at 1060 nm.

### 3.3 Monitoring cataract formation in mice

The opacification process of the murine crystalline lens triggered by anesthesia was continuously imaged with OCT. Sequential volumetric data sets were acquired every two minutes after the mouse was anesthetized and aligned, which took ∼10 minutes. The imaging time was limited to 30 minutes for each eye. When we performed the wavelength comparison, we realized that although switching between both modalities was rather quick (about 6 seconds), the mouse eye had to be realigned because of a slight focal shift of the sample arm optics for each wavelength and slow drift of the mouse eye over the course of the experiment. To avoid delays in the acquisition of quickly repeated volumes during cataract formation, we opted for using only one of the two OCT subsystems (i.e. either only 1060 nm or only 1310 nm) per eye when investigating the opacification formation in vivo. After the time course for a given eye was acquired, the OCT subsystems were switched and a final scan of the same eye was acquired with the new wavelength before switching eyes.

**Fig. 5.**
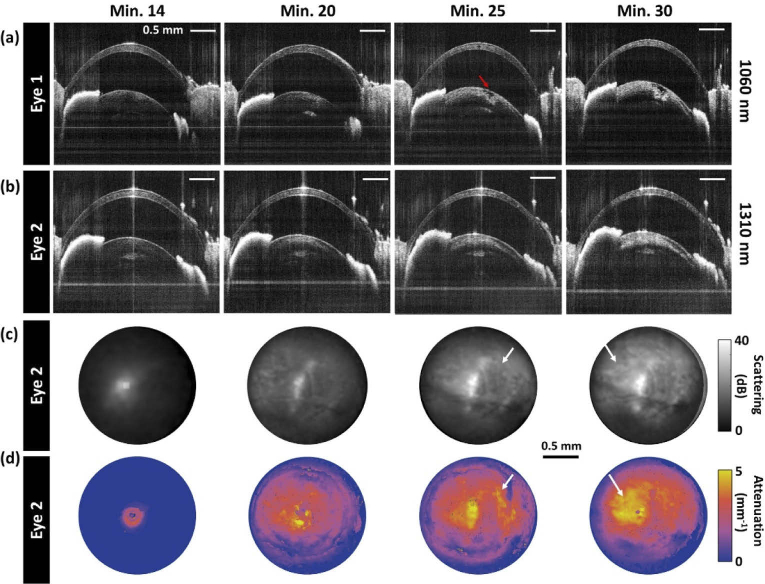
Anesthesia-induced cataract development investigated with OCT at (a) 1060 nm and (b) 1310 nm. Sequences of B-scan images are shown for a time frame from 14 to 30 minutes after the induction of anesthesia. The respective sequence of AIP maps at 1310 nm can be observed in panel (c) where the scattering increased with cataract severity. The corresponding attenuation map progression is shown in panel (d). Two locations with higher attenuation but low scattering signal are indicated with arrows.

Figure 5 shows the cataract formation process of two different eyes imaged with each one of the OCT subsystems. Figure 5(a) shows the development of an anesthesia induced opacification in the mouse lens cortex imaged at 1060 nm. When no cataract had formed yet, only a very weak OCT signal of the lens structure was observed beneath the surface, as can be seen in the first image acquired at the beginning of the experiments. A small scattering feature in the lens was visible 20 minutes after anesthesia was administered, however as the cataract progressed, a severe lesion appeared in the cortex and prevented the light to reach deeper areas of the lens as indicated by the red arrow. Figure 5(b) shows another eye imaged at 1310 nm where the scattering signal intensity in the lens cortex increased over time as the cataract evolved. This can be also seen in the sequence of the respective average scattering maps shown in Fig. 5(c). The attenuation maps of this lens shown in Fig. 5(d) reveal an increase of the attenuation over time in parallel with the lens opacification. Note that the scattering and attenuation characteristics are similar but not identical for all regions in these maps such as the areas indicated by arrows in Fig. 5(c,d).

### 3.4 Retinal projection and cataract severity

**Fig. 6.**
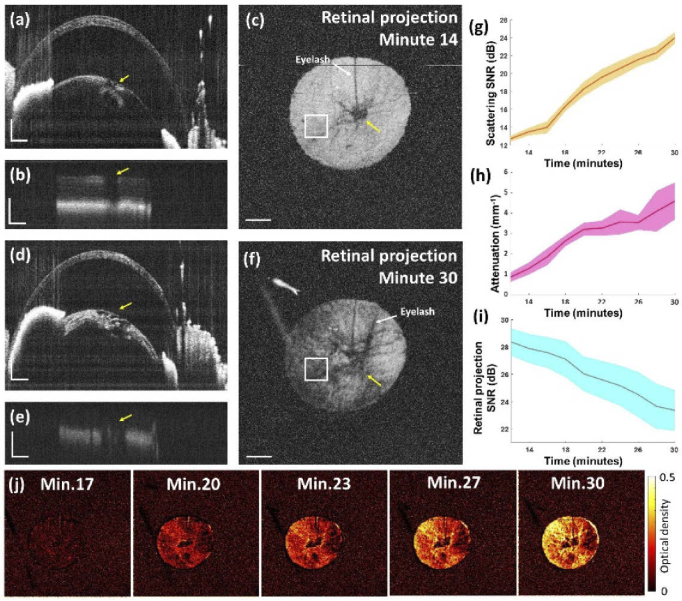
Retinal projection intensity and cataract severity. (a) Anterior segment B-scan acquired at the beginning of the imaging session where a bubble-shaped lesion is indicated by a yellow arrow. (b) Cropped retinal projected region of interest from the same B-scan. Good quality of details can be observed except in the area beneath the bubble lesion indicated by the yellow arrow. (c) Retinal projection map of the same eye. A strong and homogeneous signal can be observed over the entire area except at the location of the lesion. (d) Anterior segment B-scan of the same eye 16 minutes later when an opacification had formed in the cortex of the lens. (e) The light projected at the retina of the corresponding time point shows a decreased image quality and lower intensity. (f) The respective retinal projection clearly shows lower intensity with much poorer image quality. (g) Plot of the progression of the scattering SNR values over time in the lens region corresponding to the area indicated by a square. Line is mean value and shading is standard deviation. (h) Plot of the progression of the attenuation values in the crystalline lens calculated in the same area. (i) Plot of the SNR of the retinal projection extracted of the same region of interest over time. (j) Full field optical density maps calculated with respect to the retinal projection intensity measured at the first measurement done at minute 14. Scale bars correspond to 0.2 mm. Note that the scale bars and region of interest of the retinal projection maps do not correspond to an area of the retina but to the scanned anterior segment area. A time-lapse of the given maps can be seen in Visualization 1.

In order to directly assess how much light can reach the retina depending on the grade of opacity in the crystalline lens and thus quantify the loss of light available for vision, the retinal projection was calculated from the 1310-nm OCT data (given the higher penetration this sub-system provided) during in vivo cataract formation in the imaged eyes. Figure 6(a) shows the OCT B-scan of a mouse anterior segment at the beginning of the imaging process where most of the lens appears clear except for a bubble-shaped lesion adjacent to the cortex indicated by a yellow arrow. Figure 6(b) shows the same B-scan cropped to the deep range (z = 4.25–5 mm, in air) including the retina. Most retinal layers can be clearly seen despite not being in the focus, especially on the left side of the image which is not shaded by opacifications in the lens. However, the location of the retina corresponding to the lens lesion marked by the yellow arrow shows a decrease in intensity, indicating that the lesion is partially blocking light from reaching the posterior parts of the eye, in particular the retina. This can be observed even better in the retinal projection map shown in Fig. 6(c) where a rather homogeneous retina can be seen except for some locations shaded by lesions and eyelashes. Figure 6(d) shows the B-scan of the same eye location imaged 16 minutes later where a severe cataract has formed. Here, not only has the lesion area worsened, but also the rest of the visible part of the lens cortex has been invaded by an opacification. The corresponding retinal image shown in Fig. 6(e) exhibits a decreased image quality and intensity. Figure 6(f) shows the corresponding retinal projection with a structured pattern of greatly reduced signal intensity. The temporal evolution of the scattering signal during the course of cataract formation was extracted from the lens at the location indicated by white squares in Figs. 6(c,f) and is shown in Fig. 6(g). The respective evolution of the attenuation coefficient is plotted in Fig. 6(h). An increase in scattering and attenuation over time related to the process of cataract formation is observed. The progression of the retinal projection SNR value at the region of interest is shown in Fig. 6(i). A decrease of up to 4 dB of retinal SNR can be observed as the cataract develops. By taking an area of the retinal projection at the beginning of the imaging session where no opacifications were observed as a reference of full vision quality, an optical density of 0.42 was measured for the cataract at the region of interest by the end of the formation process. Figure 6(j) shows a full field of view of the optical density over time with respect to the first measurement. A time-lapse showing the evolution of the lens AIP, attenuation coefficient and retinal projection maps during the formation of the cataract in the mouse eye can be seen in Visualization 1.

**Fig. 7.**
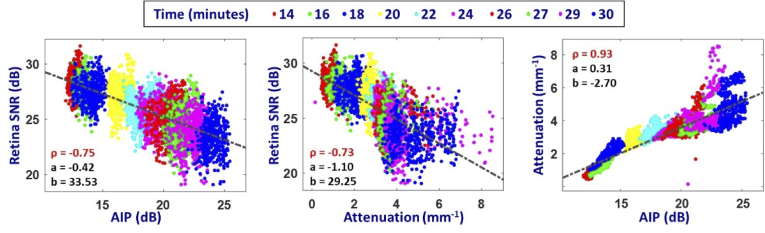
Correlation plots and Spearman correlation coefficient between lens AIP SNR, lens attenuation coefficient and retinal projection SNR during the cataract formation. Each individual point of the correlation plot corresponds to a single pixel in the respective OCT maps. The different color clusters of the correlation plots correspond to a different acquisition time point ranging from 14 to 30 minutes after anesthesia injection.

The pixel-wise correlation between SNRs of the image intensity data from the lens, the attenuation coefficients measured in the evaluation slab in the anterior lens, and the SNRs measured in the retinal projection in the selected ROI was calculated. Figure 7 shows the correlation plots between the three pairs of variables with the estimated coefficients of the linear regressions of type Y = a*X + b and the corresponding Spearman’s correlation coefficients (ρ). A strong correlation (ρ = 0.93, p < 0.001) was observed between the AIP and the attenuation coefficient measured in the crystalline lens. A moderate negative correlation was observed both between the retinal projection SNR with respect to the scattering AIP (ρ = -0.75, p < 0.001) and the lens attenuation coefficient (ρ = -0.73, p < 0.001).

## 4. Discussion

With the advantage of providing volumetric visualization of the ocular morphology, OCT has proven to be a powerful tool for volumetric imaging of cataracts in both humans and animal models [30–34]. However, the quantitative assessment of lens status using OCT for objective diagnosis still lacks an established scheme that provides a clear and accurate readout for clinicians and researchers. Visual observation with OCT has been used to provide the location and size of the cataract and has been successfully correlated to the LOCS III standard [26,33]; however, this rather qualitative method can be influenced by examiner interpretation and falls short of providing quantitative values. Since OCT detects the backscattered light from tissues, the relative intensity of the lenticular OCT signal has also been proposed as a quantitative parameter for measuring the scattering properties in the lens and has proved effective in differentiating the severity of the opacification [31,33]. However, in cases where strong opacifications form in the anterior part of the lens, light may be blocked from reaching the posterior parts of the lens, leading to a misinterpretation because opacifications at greater depths cannot be observed.

In this work, we have not only used OCT intensity data, but also mapped the global attenuation coefficient of cataractous regions in the lens. Higher attenuation values on the order of 5.0 ± 0.8 mm^-1^ were observed in eyes with cataract versus 1.0 ± 0.2 mm^-1^ in non-cataractous eyes. We have demonstrated that scattering and attenuation of the lens increased as the lens became opaque during the anesthesia-induced cataract formation process. Although the scattering signal and attenuation measured in the lens followed the same tendency during the formation of cataracts and are strongly correlated as shown in Fig. 6–7, some small areas of the anterior cortex also showed higher attenuation coefficients of 4.4 ± 0.3 mm^-1^ but did not exhibit higher SNR as indicated in Fig. 5, suggesting that scattering and µ_a_ may be decoupled in some cortical cataracts. This may be explained by the fact that at a microscopic level, cataracts do not only manifest themselves as increased scattering, but also in other forms of opacification such as vacuoles, water clefts or cortical spokes [31]. Hence, the use of both parameters together can provide a more complete analysis of the optical characteristics of cataracts. The attenuation coefficient might be a good candidate for staging the cataract severity based on the fact that the more severe the cataract the more it will attenuate the light as shown sections 3.3 and 3.4. For this work, we decided to extract the global attenuation from the OCT signal by using the curve fitting method based on a single scattering model [49,52–54]. One limitation of this simplified method is that it requires several data points with sufficiently high SNR. The inclusion of many pixels from the noise floor (e.g. when an improper window length is used or no cataract is present) can distort the fit and thus falsify the results. For our evaluation (section 2.3), an axial window length of 350 µm was chosen for the exponential fit and a constant attenuation coefficient was considered for the whole axial length. The use of a depth-resolved method however might provide a more precise quantification since axial regions with stronger cataract are expected to have a higher attenuation coefficient. In addition, the confocal point spread function and the roll-off can affect the OCT signal intensity and therefore influence the accuracy of attenuation coefficient estimation [55,56]. The murine lens follows a gradient refractive index profile similar to that of humans, however the values reported in the literature vary slightly depending on the mouse model and technique employed [57,58]. Therefore, for this work, a constant refractive index has been assumed for the entire mouse lens according to the work of Chakraborty et al. [46]. In future work, a more elaborate parameter extraction using a depth-resolved method that accounts for a gradient refractive index will be implemented and point spread function (PSF) modeling will be included. The images shown in this manuscript were not corrected for refraction of the ocular structures, however the implementation of refraction correction algorithms in the future could enable to obtain lens biometrics during the cataract development.

We have investigated the wavelength dependence of OCT imaging performance for cataract investigation by comparing image data acquired in two different wavelength bands commonly used for anterior segment imaging [27], namely 1060 nm and 1310 nm. A common sample arm was used to keep the imaging conditions as close as possible between both wavelengths and similar illumination power was used. However small differences in the refractive properties of the system components when the two beams passed through the same optical elements resulted in slightly different system parameters. While both wavelengths performed similar when imaging the opacifications in the mouse eye, a slightly higher and more homogeneous scattering signal was observed from the crystalline lens using 1060-nm OCT. However, the 1310-nm source provided deeper penetration and thus better access to the posterior parts of the eye such as the posterior lens surface and the retina in small animal models. Shorter wavelengths in OCT typically give better tissue contrast; this is more pronounced when operating at even shorter wavelength bands such as 840 nm [40,41,62,63]. In general, the two candidates used in this study provided good performance for cataract investigation and allow the use of higher power levels without compromising health safety [57].

When OCT is used for ophthalmological applications, two different regions are usually investigated, namely the anterior segment and the retina [27,65]. Depending on the ocular region of interest, different optical scanning schemes have to be used: a fan sweep scan with a collimated beam to image the retina or a telecentric scan using a focused beam for the anterior segment. It is obvious that to investigate cataracts, one must focus on studying the crystalline lens, however the visual acuity is ultimately determined by the image formed on the retina. Some studies have reported how cataracts influence image quality when investigating the retina [66–68], however these studies considered cataracts as a drawback when performing retinal investigations. In this work, we suggest a new perspective using quantitatively assessed retinal image quality as a method for measuring and mapping opacifications in the crystalline lens. The intensity of the retinal projection signal has been used for mapping the effect of cataracts in the anterior segment which enabled a direct correlation with opacification severity. This method has the limitation that the map of light intensity projected to the retina is not only indicative of problems in the lens. Any abnormal features in other parts of the anterior segment, such as the cornea, or even within the retina itself will also affect light transmission and therefore show up on the retinal projection map. However, the benefit of having a long imaging range that includes both the retina and anterior segment enables us to compare the retinal projection mapping with scattering and attenuation quantification of the crystalline lens simultaneously and therefore provide a better overall interpretation of the cataract.

The cataracts investigated in this paper were induced by anesthesia effects in the mice [50,51]. They developed rapidly within the first ten minutes after narcotic injection. As immobilization and alignment of the mouse eye took some time, partial opacifications were sometimes already present at the start of the imaging procedure. In future experiments, it will be interesting to use a mouse model that naturally develops congenital cataracts and hence allow us to perform a longitudinal study over a much longer time period [69]. Other contrast methods such as polarization-sensitive OCT have been shown to provide an additional contrast channel and thus might also provide better insights of the cataract formation process and help in the diagnosis [70,71] when combined with the methods demonstrated here.

## 5. Conclusion

In conclusion, we have demonstrated the use of OCT for investigating depth-resolved scattering and average anterior cortical attenuation properties of cataracts in the murine crystalline lens. Our results revealed a correlation of scattering SNR and attenuation coefficient values with cataract severity, as well as a decrease in retinal projection intensity when a strong opacity was formed in the lens. Two different wavelength bands centered at 1060 nm and 1310 nm were tested for cataract imaging with OCT, indicating that 1060 nm may be more beneficial when targeting lens scatter, while 1310 enabled deeper penetration through cataracts to the retina. We have also introduced a novel method for cataract quantification based on the retinal projection intensity. The combination of the different quantification methods presented here with the volumetric and non-invasive benefits that OCT provides suggest that this technique might be an interesting candidate for performing longitudinal eye studies and cataracts research.

## Data Availability

Data related to the results presented in this paper can be obtained from the authors upon reasonable request.
